# *Helicobacter Pylori*-Negative MALT Lymphoma: A Series of Two Cases Presenting with Life-Threatening Upper Gastrointestinal Bleeding

**DOI:** 10.1155/2023/8244696

**Published:** 2023-03-23

**Authors:** Seo Hyun Kim, Youssef Soliman, Vikas N. Chitnavis, Maithili V. Chitnavis

**Affiliations:** ^1^Virginia Tech Carilion School of Medicine, Roanoke, VA, USA; ^2^Gastroenterology, City of Hope Phoenix, Goodyear, AZ, USA; ^3^Department of Internal Medicine, Division of Gastroenterology, Carilion Clinic and Virginia Tech Carilion School of Medicine, Roanoke, VA, USA; ^4^Inflammatory Bowel Disease Section, Atrium Health Gastroenterology, Charlotte, NC, USA

## Abstract

Mucosa-associated lymphoid tissue (MALT) lymphoma is a common cause of gastric lymphoma. Although most cases are associated with an *H. pylori* infection, approximately 10% are *H. pylori*-negative. Patients with gastric MALT lymphoma are usually asymptomatic or present with nonspecific symptoms such as abdominal pain, dyspepsia, weight loss, and occult gastrointestinal bleeding. In this report, we describe two patients with *H. pylori*-negative MALT lymphoma who both presented with acute upper gastrointestinal bleeding that led to hemodynamic instability. After resuscitation, emergent endoscopy was performed. Both patients had the *t* (11; 18) (q21; q21) translocation, which prompted direct treatment by radiotherapy.

## 1. Introduction

MALT lymphoma is an extranodal non-Hodgkin lymphoma that most commonly affects the stomach. While approximately 90% of gastric MALT lymphomas are associated with an *H. pylori* infection, approximately 10% are *H. pylori*-negative [1]. Patients with gastric MALT lymphoma are usually asymptomatic or have nonspecific abdominal symptoms [2]. Hematemesis and hemodynamic instability are rare and are scarcely reported in the literature. We report a unique series of two cases of *H. pylori*-negative MALT lymphoma that presented with upper gastrointestinal hemorrhage requiring urgent resuscitation and endoscopy. These cases highlight the importance of recognizing upper gastrointestinal hemorrhage and hypovolemic shock as potential presentations of *H. pylori*-negative gastric MALT lymphomas in order to achieve timely intervention.

## 2. Case Report

### 2.1. Case 1

A 73-year-old female with history of sick sinus syndrome status-post pacemaker implantation and anxiety presented with generalized weakness, fatigue, and postprandial abdominal pain for five days. Associated symptoms included near-syncope, diaphoresis, nausea, abdominal bloating, and melena. She denied NSAID, anticoagulation, tobacco, alcohol, or drug use. She had no family history of gastrointestinal diseases or cancers.

The patient's blood pressure was 90/58 mmHg, pulse 85, respiratory rate 18, and temperature 97.1 degrees F. Physical exam was unremarkable except for melena on rectal exam. CBC revealed a hemoglobin of 9.6 g/dL (baseline: 13.0 g/dL) and was otherwise normal. CMP revealed an elevated BUN/Cr ratio of 42.9 but was otherwise unremarkable. Coagulation parameters were normal. Troponin was normal. Pro-BNP was elevated at 337.4 pg/dL (normal: <125 pg/dL). Chest X-ray was negative for acute processes.

**Figure 1:**
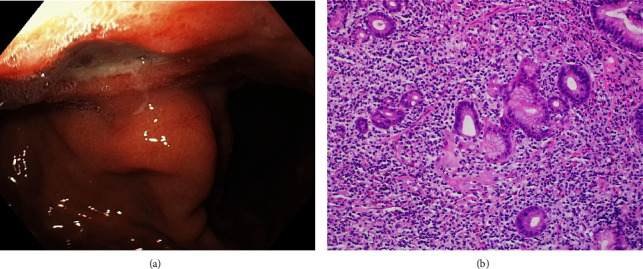
Endoscopic image of gastric ulcer in first case (a). Microscopic image H&E stained 400× of lymphoepithelial lesion with surrounding lymphocyte mucosal invasion (b).

After resuscitation with intravenous (IV) fluids and IV pantoprazole, an esophagogastroduodenoscopy (EGD) was notable for several clean-based gastric body ulcers, the largest being 1.5 cm (Figure 1(a)). Gastric biopsies were consistent with atypical B-cell predominance in the lamina propria and muscularis mucosa (Figure 1(b)). No *H. pylori* was detected by biopsy and fecal antigen test. FISH demonstrated rearrangement of the *MALT1* gene *t* (11; 18) (q21; q21) in 57% of interphase cells, consistent with MALT lymphoma. Whole-body imaging revealed no metastases. She was referred to Radiation Oncology and underwent radiotherapy with a total dose of 30 Gy over 27 days. Remission was achieved and sustained on EGD at 3, 6, and 9 months.

### 2.2. Case 2

A 91-year-old female with history of coronary artery disease status-post percutaneous coronary intervention on dual-antiplatelet therapy, hypertension, GERD, and breast cancer status-post mastectomy presented with chest pain and hematemesis. Associated symptoms included nausea, lightheadedness, and dizziness. She denied melena or hematochezia. She denied NSAID, tobacco, alcohol, or drug use. She had no history or risk factors for cirrhosis. There was no family history of gastrointestinal disease or cancers. Surgical history included cholecystectomy and total abdominal hysterectomy several years prior.

At presentation, the patient's blood pressure was 71/47 mm Hg, pulse 77, respiratory rate 32, and temperature 97.7 degrees F. Physical exam was unremarkable. CBC revealed hemoglobin of 8.5 g/dL (baseline: 12.3 g/dL). CMP showed a BUN/Cr ratio of 71, but other parameters including liver enzymes were normal. Coagulation parameters were normal. EKG and troponin were normal. Chest X-ray was negative for acute processes. CTA of the chest, abdomen, and pelvis showed mild diverticulosis and signs of intraluminal IV contrast extravasation in the distal duodenum.

**Figure 2:**
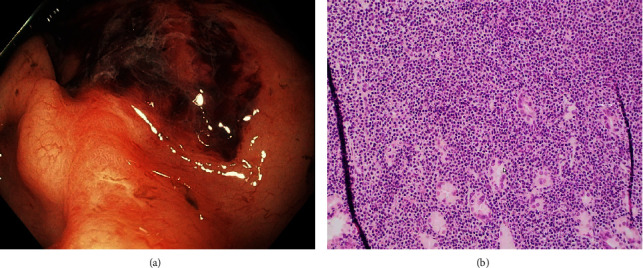
Endoscopic image of polypoid lesion in second case (a). Microscopic image H&E stained 200× of diffuse lymphocyte invasion into the lamina propria and muscularis mucosa (b).

After resuscitation with IV fluids and transfusion with two units of packed RBCs, an EGD was performed. This was notable for a subtle, polypoid lesion in the gastric body along with oozing and large amounts of blood clots (Figure 2(a)). Saline-assisted snare biopsy revealed infiltration with B-cell lymphocytes into the muscularis mucosa (Figure 2(b)). No *H. pylori* was detected by biopsy and fecal antigen test. FISH demonstrated rearrangement of the *MALT1* gene *t* (11; 18) (q21; q21) in 72% of interphase cells. Whole-body PET/CT imaging revealed no metastases. She is planned to complete targeted radiotherapy with a total dose of 24–30 Gy.

## 3. Discussion

First described by Isaacson and Wright in 1983, MALT lymphoma is a low-grade extranodal non-Hodgkin lymphoma [3]. The most common site of MALT lymphoma is in the stomach, although it can also arise in the ocular adnexa, thyroid, and small intestine [4]. In the United States, MALT lymphoma accounts for approximately 8% of non-Hodgkin lymphoma, but makes up about 50% of lymphomas in the stomach [5].

Patients with gastric MALT lymphoma are asymptomatic or present with nonspecific symptoms such as epigastric abdominal pain, dyspepsia, anorexia, weight loss, and occult gastrointestinal bleeding [2]. Hematemesis and hemodynamic instability are rare. Harne et al. [6] is the only other case report of *H. pylori*-negative MALT lymphoma that presented similarly to our two patients with upper gastrointestinal bleeding and hypovolemic shock. Their case had revealed erythematous, friable mucosa on EGD, whereas our cases uniquely showed bleeding ulcers (Case 1) and an abnormal polypoid lesion (Case 2). The lesions of gastric MALT lymphoma are often detected by EGD and can resemble other gastric lesions such as gastritis, polyps, ulcers, or masses [7]. Definitive diagnosis is made by histopathology.

Approximately 90% of gastric MALT lymphomas are associated with an *H. pylori* infection, while about 10% are *H. pylori*-negative [1]. The pathogenesis of *H. pylori-*negative MALT lymphoma is poorly understood, but may arise due to chronic infection by viruses or bacteria other than *H. pylori* [8]. Molecularly, gastric MALT lymphomas involve genetic translocations that activate the NF-B pathway, with the most common being *t* (11; 18) (q21; q21) which results in an API2/MALT1 fusion gene. Interestingly, this translocation is more frequently detected in *H. pylori*-negative MALT lymphomas [9].

While *H. pylori* eradication therapy is first-line for *H. pylori-*positive MALT lymphomas, empiric antibiotic therapy only has a 28% response rate in *H. pylori*-negative MALT lymphoma compared to 75% in *H. pylori*-positive cases [10]. Studies have shown that patients with the *t* (11; 18) (q21; q21) translocation are particularly less responsive to antibiotics compared to patients without the translocation [11, 12]. For this reason, both of our patients were directly treated with radiotherapy. Radiotherapy has been associated with high clinical remission rates, low toxicity profile, and long-term remission.

Interestingly, both patients in our report had a past history of heart disease. Although there are reported cases of MALT lymphomas invading the cardiac wall (e.g., primary epicardial MALT lymphoma) [13], there is paucity of the literature discussing an association between gastric MALT lymphomas and heart disease. Furthermore, both of our patients had a relatively low heart rate despite their low blood pressures. While it is unclear why Patient 1 had a relatively low heart rate, Patient 2's low heart rate can be explained by the fact that the patient was on metoprolol tartrate, a heart rate-lowering agent.

We describe two unusual cases of *H. pylori*-negative gastric MALT lymphomas that presented with life-threatening upper gastrointestinal bleeding and hemodynamic instability. Given the scarce reports of this in the literature, it is critical for providers to recognize these acute signs as a potential initial presentation of gastric MALT lymphoma and to keep a broad differential.

## Data Availability

The clinical data used to support the findings of this study are included within the article.
